# The forerunners and successful partnerships behind the BioNTech mRNA vaccine

**DOI:** 10.1007/s13353-023-00793-5

**Published:** 2023-10-20

**Authors:** Ilkin Aygün, Jan Barciszewski

**Affiliations:** 1 Institute of Bioorganic Chemistry, Polish Academy of Sciences, Z. Noskowskiego, 12/14, 61-704 Poznan, Poland; 2 NanoBioMedical Center, Adam Mickiewicz University, Poznan, Poland

**Keywords:** COVID-19, mRNA technology, Vaccine development, BioNTech, Pandemic

## Abstract

The discovery of nucleic acids stands as a paramount achievement in the history of scientific endeavors. By applying transformative advancements in the fields of chemistry and physics to biological systems, researchers unveiled the enigmatic nature of life. Notably, messenger RNA (mRNA) emerged as a crucial player in this profound revelation, serving as a transient intermediary for genetic information transfer between genes and proteins. Groundbreaking investigations carried out from 1944 to 1961 led to the initial identification of this pivotal molecule, captivating scientific interest for the past three decades. The field of mRNA research has witnessed a transformative shift owing to the development of cap analogs and nucleotide modifications. This revolutionary progress has fostered a new generation of potent therapeutics. Prior to the advent of the coronavirus pandemic, numerous scientists had already begun exploring the unique properties of mRNA. However, with the onset of the pandemic, mRNA catapulted into the limelight as a heroic agent, providing the foundation for highly effective vaccines that have played a crucial role in mitigating the spread of severe acute respiratory syndrome coronavirus 2 (SARS-CoV-2). The successive generations of cap analogs have significantly enhanced the translation efficacy of mRNA, while the discovery of suitable purification, packaging, and delivery methods has paved the way for groundbreaking medical breakthroughs. Pioneers in the field such as Katalin Karikó, Drew Weissman, Edward Darzynkiewicz, Robert Rhodes, Ugur Sahin, and Ozlem Tureci have made significant contributions during the early stages of mRNA research, warranting acknowledgement for their visionary endeavors. The narrative of mRNA represents a remarkable journey marked by a succession of breakthroughs in a discipline that holds immense promise for the future of medicine. Thanks to the pioneering work of these exceptional scientists, we are well-positioned to unlock the full potential of this extraordinary molecule, ushering in a new era of medical advancements.

## Introduction

The emergence of the coronavirus disease 2019 (COVID-19) pandemic necessitated urgent efforts to develop effective vaccines. In this endeavor, mRNA technology has emerged as a groundbreaking tool in vaccine development. Utilizing mRNA to encode specific viral antigens and trigger an immune response, Moderna, BioNTech, and CureVac made significant strides in combating COVID-19. This text explores the transformative journey of mRNA technology, from its discovery to its pivotal role in creating COVID-19 vaccines. The work of visionary scientists, including Ugur Sahin, Ozlem Tureci, Katalin Karikó, Drew Weissman, Edward Darzynkiewicz, and Robert Rhodes, played a crucial role in advancing mRNA’s potential by modifying caps and stabilizing molecules, leading to enhanced translational efficiency and reduced immunogenicity. Beyond the pandemic, mRNA research holds immense promise for developing vaccines against other infectious diseases and treating genetic disorders. This text highlights the power of collaboration and scientific innovation, paving the way for a bright future in medicine through harnessing mRNA’s full potential.

## Coronavirus breakthrough on mRNA vaccine development

SARS-CoV-2 is a virulent pathogen with an RNA genome that belongs to the family of coronaviruses. This highly infectious virus has spread rapidly across the globe, causing the emergence of the COVID-19 pandemic. Its single-stranded RNA genome is encoded with genes that express several viral proteins, including the spike protein, which facilitates viral entry into host cells. The resulting infection can cause a range of symptoms, including coughing, fever, chest discomfort, and, in severe cases, acute respiratory distress syndrome (ARDS), which may lead to multi-organ failure and death (Hu et al. 2021; Sun et al. 2020). According to the latest available data, as of July 19, 2023, the total number of confirmed cases of COVID-19 globally amounted to 768,237,788. This staggering figure underscores the immense scale of the pandemic and its far-reaching impact on global public health. Moreover, the reported fatalities attributed to viral infection or related complications currently stand at 6,951,677. These numbers serve as a stark reminder of the ongoing threat posed by COVID-19 and the urgent need for continued vigilance, research, and mitigation strategies to protect the health and well-being of people worldwide (John Hopkins Coronavirus Resource Center, n.d.). Developing safe and effective vaccines to contain the COVID-19 pandemic has been a top priority for scientific organizations and pharmaceutical companies worldwide (Krammer 2020).

## mRNA vs traditional vaccines

**Fig. 1:**
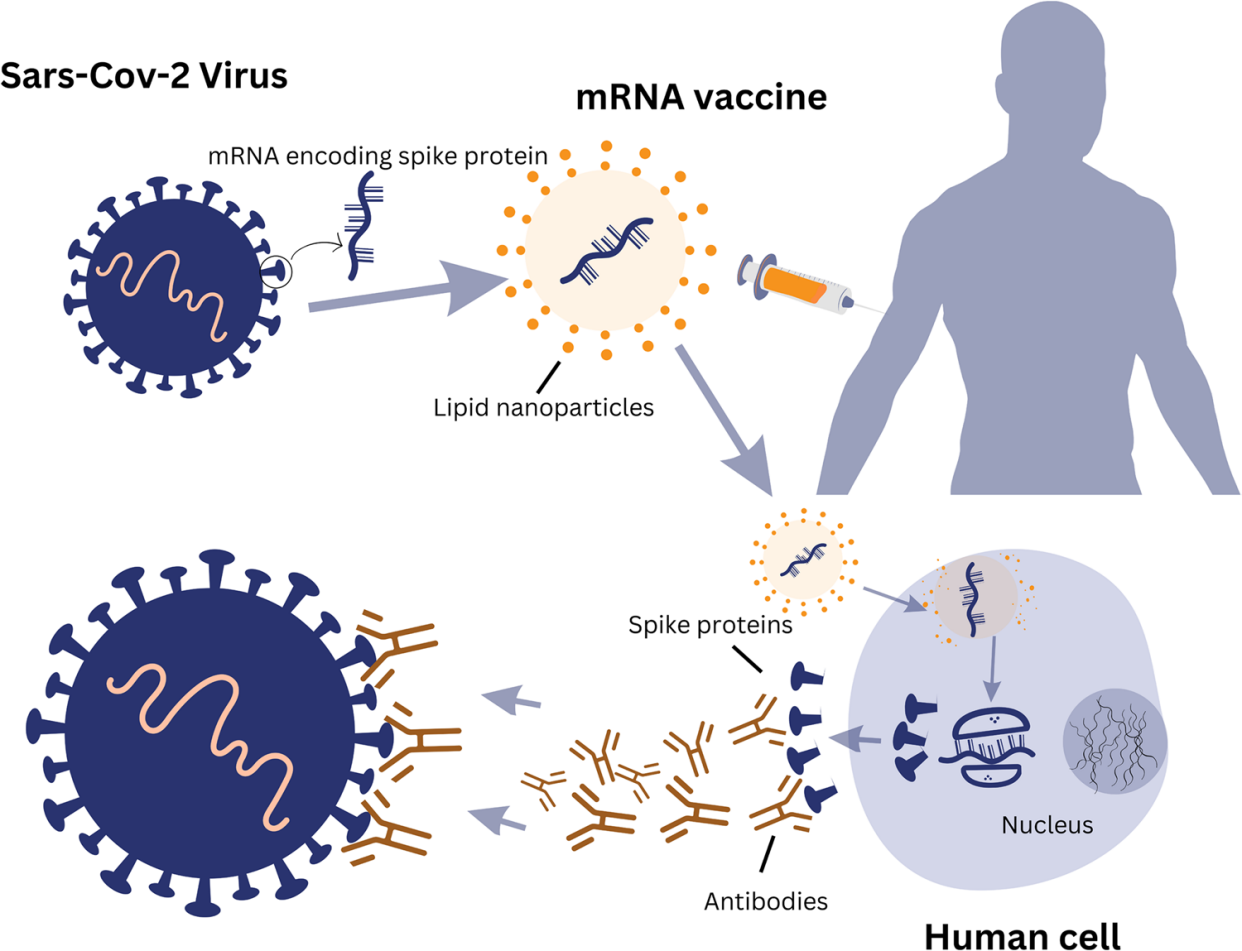
Schematic representation of the mechanism of action of mRNA vaccines. This figure illustrates the mechanism of action of mRNA vaccines. Following injection, mRNA within the vaccine is taken up by host cells, instructing them to synthesize the viral spike protein. Displayed on cell surfaces, this protein triggers an immune response, characterized by antibody production. These antibodies can neutralize the virus upon future exposure. Subsequently, the transient mRNA is enzymatically degraded and removed from the body

The use of mRNA technology to encode viral antigens in vaccine development represents a major advancement in the field of immunology. Traditionally, vaccine research entails immunizing with a weakened virus or a non-infectious antigen produced from a viral protein. The use of mRNA in vaccine development represents a significant departure from these conventional procedures. The mRNA-based vaccine technology involves delivering genetic material that encodes a specific antigen from the virus to cells in the human body. Once inside the cells, the mRNA is used as a blueprint to produce the antigen, which then triggers an immune response (Fig. 1). The use of mRNA technology in vaccine development has several advantages over traditional vaccine development. Firstly, mRNA-based vaccines can be manufactured more rapidly and with higher efficiency levels than traditional vaccines. Secondly, mRNA vaccines can be easily modified to respond to new virus strains or emerging infectious diseases. Thirdly, mRNA-based vaccines have an excellent safety profile and do not contain any live viruses or infectious genetic material.

## Comparing mRNA COVID-19 vaccines: technology and success

The mRNA vaccine story began in January 2020, when Chinese scientists first shared the genetic sequence of SARS-CoV-2, the virus responsible for COVID-19. The development of mRNA vaccines has been a culmination of over 30 years of research. Although the technology has been studied for decades, it was only during the COVID-19 pandemic that the potential of mRNA vaccines was fully realized. Tens of mRNA vaccine candidates from various countries were investigated since 2020 during the SARS-CoV-2 pandemic, and millions of people are currently vaccinated with mRNA-based vaccines that have been approved for immediate use. This suggests that the SARS-CoV-2 pandemic will serve as a turning point in the future for both vaccines and mRNA-based therapeutic approaches. Ongoing research in mRNA technology holds immense potential in not only developing effective vaccines against other infectious diseases but also treating various genetic and rare disorders. It is likely that the success of mRNA vaccines against COVID-19 will pave the way for the development of new vaccines and therapies that can target a range of diseases and medical conditions.

Three teams — Moderna, BioNTech, and CureVac — have utilized this novel approach in the race to develop safe and effective vaccines to contain the COVID-19 pandemic. However, due to the utilization of unmodified RNA in CureVac, as opposed to the nucleoside-modified RNA employed in the Pfizer-BioNTech COVID-19 vaccine and Moderna COVID-19 vaccine, it exhibits a lower efficiency of only 48% and is unable to achieve the same level of efficacy as the aforementioned vaccines.

On December 23, 2021, the COVID-19 vaccine developed in collaboration with Pfizer-BioNTech became the first to receive approval from the FDA (“FDA Approves First COVID-19 Vaccine,” 2021), followed by Moderna as the second. Both the Pfizer-BioNTech and Moderna COVID-19 vaccines are composed of modified mRNA that encodes the spike protein of the SARS-CoV-2 virus. Lipids are included in the vaccines to encapsulate the mRNA, protect it from degradation, and promote efficient delivery to cells. The specific lipids used in each vaccine are different (Table 1). The vaccines also contain salts to help maintain the correct pH of the solution, and sugars to stabilize the structure of the mRNA and enhance its uptake into cells. The Pfizer-BioNTech vaccine contains an acidic buffer to maintain the pH of the vaccine, while the Moderna vaccine contains additional stabilizers to protect the mRNA during storage and transportation.

**Table 1:** Composition of the Pfizer-BioNTech, Moderna, and CureVac COVID-19 vaccines

	mRNA	Lipids	Salts	Other
BioNTech/Pfizer (BNT162b2)	Full-length spike with proline substitutions (K986P, V987P)	*Ionizable cationic lipid*	Potassium chloride, monobasic potassium phosphate, sodium chloride basic sodium, phosphate dihydrate	Sucrose
	- -*N1-methylpseudouridine*	- ALC-0315 (Acuitas)		
	- Codon optimization	*Helper lipids*		
	- GC-enriched sequence	- DSPC		
	- dsRNA removal	- Cholesterol		
	- Modified 5′ CAP1 structure (m7G + -5′-ppp-5′-Am)	- PEG-DMA		
	- 5′ UTR: human α-globin RNA with optimized Kozak sequence	Lipid molar ratios* (50:10:38.5:1.5 mol%)		
	- 3′ UTR: AES and mtRNR1 3′ UTR Motives (Moderna (n.d.))	RNA to lipid ratio*		
	- 110 Poly(A) tail with nucleotide-linker (GCAUAUGACU)	~ 0.05 (wt/wt)		
Moderna (mRNA-1273)	Full-length spike with proline substitutions (K986P, V987P)	*Lipid nanoparticle Ionizable cationic lipid*	Thromethamine, thomethamine hydrocholoride, acetic acid, sodium acetate	Sucrose
	- *N1-methylpseudouridine*	- SM-102		
	- dsRNA removal—Undisclosed structural elements	*Helper lipids*		
		- DSPC		
		- Cholesterol		
		- PEG-DMG		
		Lipid molar ratios* (50:10:38.5:1.5 mol%)		
		RNA to lipid ratio* ~ 0.05 (wt/wt)		
CureVac (CvnCoV)	Full-length spike with proline substitutions (K986P, V987P)	Lipid nanoparticle	Saline	
	- *-Unmodified nucleotide sequence engineered*	*Ionizable cationic lipid*		
	- Codon optimization	- Acuitas lipid (undisclosed) *Helper lipids*		
	- -GC-enriched sequence	- DSPC		
	- dsRNA removal	- Cholesterol		
	- Modified 5′ CAP1 structure (m7G + -5′-ppp-5′-Am)	- PEG-lipid (undisclosed)		
	- 5′UTR: Artifacts from restriction and transcription site, plus Kozak sequence	Lipid molar ratios* (50:10:38.5:1.5 mol%)		
	- -3′ UTR comprising human alpha-globin	RNA to lipid ratio*		
		~ 0.05 (wt/wt)		

The obstacles inhibiting the broad use of mRNA as a medicinal agent were overcame in part by nucleic acids’ bases modifications. They also represent the most significant occasions in the evolution of mRNA. Translational effectiveness and immunogenicity were the two most crucial factors that needed to be addressed. The most important factor in mRNA’s extraordinary success was the ground-breaking work in these areas, particularly the development of very efficient COVID-19 vaccinations. For a variety of reasons, the effectiveness of mRNA translation is crucial. Higher effectiveness results in a reduced mRNA load and a correspondingly lower immunogenicity. The ability to boost the potency of the mRNA was made possible by the convergence of independent advancements, which enabled the body to produce as much protein from the mRNA as feasible. This can be achieved by adding various 5′ and UTR, poly-A tail, structural modification of ORF or altering the chemical structure of the 5′cap. These kinds of modifications might be able to increase the mRNA’s translational efficiency or lengthen its half-life, both of which would significantly increase both translation and mRNA stability within the cell.

Despite the fact that this success has been achieved in such a short period of time, as seen, it is due to both the accumulation of years of work and a broad vision that anticipates that studies conducted in many different parts of the world can be gathered on common ground. In this review, we will focus on how BioNTech company achieved this success, the heroes behind great work, and the success they achieved through the strong collaborations that Ugur Sahin, a great scientist, envisioned and established years ago to develop mRNA vaccines. The vaccine’s creators, Ugur Sahin and Ozlem Tureci, are co-founders of the internationally renowned company BioNTech, and also, they are among the first to recognize the potential of mRNA vaccines for infectious diseases and invested heavily in research and development. However, the speedily developed vaccine was the result of an exhaustive inquiry and outstanding collaboration.

## From immunotherapy to the development of a COVID-19 vaccine: Ugur Sahin and Ozlem Tureci

Ugur Sahin was born in Hatay, Turkey, on September 19, 1965 (Migration Und Qualifikation n.d.). When he was 4 years old, he moved to Cologne, Germany, with his mother to live with his father, who worked at the Ford Motor Company factory. Western European countries at the time tended to employ foreign labor and thus exported labor from their neighbors. After finishing high school, he enrolled in the University of Cologne’s Faculty of Medicine in 1984 and graduated in 1992 (Sahin n.d.; Wrede 2020). Under the supervision of Dr. Michael Pfreundschuh, he received his PhD in immunotherapy in tumor cells from the same university in 1993 (Gelles 2020). He received a habitation in medicine and immunology in 1999 (Wrede 2020). Between 2000 and 2001, Ugur Sahin worked at Zurich University Hospital in various departments and collaborated with 1996 Nobel Prize winners Rolf M. Zinkernagel and Hans Hengartner. He became a professor at the University of Mainz, where he met his wife, Ozlem Tureci, now a Professor for Translational Oncology and Immunology at the University Medical Center Mainz (Sahin n.d.). Ozlem Tureci, co-founder of the company, was born on March 6, 1967, in Siegen, North Rhine-Westphalia, West Germany, to a Turkish family (BBC News Türkçe, n.d.) of immigrants from Istanbul (Klusmann and Schulz 2021; Oltermann 2020). In a diverse milieu, Ozlem Tureci grew up adoring the nurses at the hospital where her father worked. At Saarland University, Ozlem Tureci finished her undergraduate studies. In 2001, she moved to Mainz and she did research at the Mainz University Hospital with the goal of using altered genetic coding to combat the immune system’s ability to fight cancer. She continued her work in the company she co-founded with Ugur Sahin in 2001 and named “Ganymed.” This name was inspired by the Turkish word “ganimet,” signifying “trophy” in English (Oltermann 2020). The couple continued to collaborate so that the immune system could detect and eliminate cancerous cells as if they were viruses entering the body (Covid-19 Aşısının Arkasındaki Iki Isim: Türkiye Kökenli Uğur Şahin Ve Özlem Türeci 2020). In 2016, Ganymed, a pioneer in sensitive antibody therapies for cancer, was acquired by the Japanese pharmaceutical company Astellas. That sale, worth 420 million Euros, was the largest in the German medical industry to that point (Ganymed 2016; “Aşının fiyatı belli oldu,” n.d.). This transaction elevated the Ozlem Tureci-Ugur Sahin couple to the ranks of Germany’s wealthiest citizens (Spotlight: The Turkish-German “dream Team” Couple Behind Pfizer’s COVID-19 Vaccine 2020). BioNTech was founded in 2008 by Ozlem Tureci and Ugur Sahin, along with Austrian immunologist and oncologist Christoph Huber. Ozlem Tureci was appointed chief medical officer of the company in 2018. She is the President of the Cancer Immunotherapy Association as well. The company, which will have 1300 employees by 2020, began by developing immunotherapy cancer treatments. With the onset of the COVID-19 pandemic in 2020, the company focused on developing a coronavirus vaccine. The couple predicted that this would occur before the coronavirus epidemic became a global pandemic, and they decided to immediately begin vaccine studies using the method they had been developing for 25 years. The duo, who called an emergency meeting of the board of directors, attempted to persuade executives who believed that what happened in China would not have a global impact. Pfizer, a US pharmaceutical company that collaborated with BioNTech on the development of the vaccine, announced on November 9, 2020, that the produced COVID-19 vaccine (BNT162) achieved 90% success (Pfizer and BioNTech Announce Vaccine Candidate Against COVID-19 Achieved Success in First Interim Analysis From Phase 3 Study | Pfizer, n.d.). Ugur Sahin and his team launched “Project Lightspeed” to develop a vaccine against COVID-19 after reading about it in The Lancet (BioNTech | Fighting the Global Pandemic with Project Lightspeed, 2020).

More than 20 years ago, Ugur Sahin and Ozlem Tureci began their research. The idea of using mRNA as therapeutic cancer vaccines intrigued these cancer immunologists. Delivering mRNA into dendritic cells was one of the goals at the time because it was recognized as one of the significant breakthroughs at the time and because dendritic cells are the primary immune response stimulators. This was accomplished and detailed by other labs in the middle of the 1990s (Steinman and Witmer 1978). However, Ugur Sahin and Ozlem Tureci were keen to transfer the mRNA straight for use in vivo, shortening the depth between the processes of in vitro transcription, purification, and then injection of the mRNA. They hypothesized that mRNA delivery to lymphoid organs like lymph nodes and spleen could be able to facilitate a particularly enhanced immune response. They began testing it, transfecting the dendritic cells in vitro with mRNA and observed translation for a while, but after that the mRNA translation rapidly decreased and had relatively low translation (Holtkamp et al. 2006). However, it is crucial to induce a strong response while having the least amount of mRNA possible.

## mRNA cap modifications to improve resilience

While several well-established laboratories had been working on this project for certain time, the fruitful collaboration between Robert Rhodes and Edward Darzynkiewicz had also just started. They both worked as postdocs in Bob Schimke’s group and dedicated years to the research on mRNA cap. Organic chemist Edward Darzynkiewicz at the University of Warsaw in Poland and Robert Rhoads, professor emeritus and former head of the Department of Biochemistry and Molecular Biology at LSU Health Shreveport, led to innovations that helped stabilize the mRNA with specific modifications to its cap to make it more resilient. An antidote in the form of the anti-reverse cap analog (ARCA), a new cap analog, was published in 2001 by Darzynkiewicz and Rhoads (Stepinski et al. 2001). It was demonstrated in research of lipofection of dendritic cells that ARCA increases translation efficiency 20-fold and works in concert with poly(A) tail elongation from 64 to 100 adenosines, which further increases 35-fold, resulting in a total increase in reporter gene production of 700-fold. Rhoads and Darzynkiewicz’s mRNA-stabilizing cap structures piqued the interest of Ugur’s team since mRNA persistence in dendritic cells leads to increased translation, the production of more neoepitopes, and an increased T cell response. He got in touch with Darzynkiewicz and Rhoads in 2008 to explain the concept that would sequence all the RNA from a patient’s tumor to identify all proteins expressed, compare them to standard proteins to find aberrant proteins synthesized by the cancer cells, create synthetic mRNA encoding these neoepitopes, and then inject it into patients where it would be taken up and translated by immature dendritic cells, thence activating T cells that would attack the patient’s cancer cells. Robert Rhodes and Edward Darzynkiewicz, who were astonished by the concept, began a fruitful collaboration with Ugur Sahin. Phosphonothioate cap analogs based on the ARCA concept were patented and acquired exclusively by BioNTech, for use in their research (Kuhn et al. 2010) not long after Ugur Sahin had established the business. This was done in order to achieve the goal of RNA-based cancer immunotherapy. Ugur Sahin has an ability for putting the pieces of the puzzle together and has always had a keen eye for the big picture. His broad vision allowed him to work primarily with mRNA heavyweights Edward Darzynkiewicz and Robert Rhodes, and he then took another significant step by hiring Katalin Karikó at BioNTech.

## Overcoming immunogenicity challenges in mRNA-based treatments

In addition to issues with translation efficacy, mRNA immunogenicity makes it challenging for mRNA-based treatments to be quickly translated into clinical practice. However, the subsequent amazing assiduity of Katalin Karikó and Drew Weissman gave crucial understanding into the fact that the majority of natural mRNA molecules include changed nucleotides, which may protect them from the cellular innate immune system. Katalin Karikó, a Hungarian scientist, received her PhD in biochemistry. She moved to the USA in 1985 as a postdoctoral researcher at Temple University in Philadelphia, where she started her journey in trying to adapt mRNA to therapeutic use, conducting clinical studies on triggering interferon production with double-stranded RNA. As a research assistant professor at the University of Pennsylvania School of Medicine, she focused her efforts on treating acute diseases with in vitro-synthesized mRNAs encoding therapeutic proteins but at that time she failing to win grants and was forced to move from lab to lab, going wherever she could find someone willing to fund her research. A chance interaction with a recently hired immunologist Drew Weissman in 1997 at the copy machine marked a turning point in the mRNA story (Yu 2021), and Katalin Karikó developed the “Revolutionary mRNA Technology Inside COVID Vaccines” (2021). Katalin Karikó and Drew Weissman learned they had a passion for the delivery of synthetic mRNA to produce highly targeted therapeutic proteins while working together at the University of Pennsylvania School of Medicine. Although research on utilizing mRNA to deliver therapeutic proteins had been ongoing for years in numerous labs, Katalin Karikó and Drew Weissman’s complementing abilities and knowledge led to the breakthrough that sped up the development of a SARS-CoV-2 vaccine. Katalin Karikó and Drew Weissman concentrated on using exogenous mRNAs delivered into cells in culture using cationic lipids to direct the creation of therapeutic proteins. They faced significant challenges since synthesized mRNA induced the production of pro-inflammatory cytokines in human dendritic cells of the innate immune system (Ni et al. 2002). However, they made a crucial observation: naturally occurring RNAs, particularly transfer RNAs (tRNAs), which they used as a control in their experiments, did not trigger the release of proinflammatory cytokines in the same way as synthetic RNAs did (Koski et al. 2004). It is well known that tRNAs contain a high — up to 25% percentage of modified nucleosides. When Katalin Karikó and Drew Weissman learned that modified nucleosides might conceal RNA from the innate immune system, it hit them like a ton of bricks. In 2005, Karikó and Weissman showed that the generation of proinflammatory cytokines by dendritic cells originating from monocytes was decreased by mRNAs produced in vitro utilizing T7 RNA polymerase and a wide range of modified nucleotide substrates (Karikó et al. 2005). Notably, the ability of mRNA to stimulate primary, blood-derived dendritic cells was eliminated by uridine derivatives. Immune stimulation by mRNA was suppressed proportionally to the number of altered nucleosides present in the mRNA, but even a few modified nucleosides had a suppressive effect. Katalin Karikó and Drew Weissman later demonstrated that incorporating pseudouridine not only reduced immunogenicity but also significantly increased translation of synthetic mRNAs in vitro and in mice (Karikó et al. 2008). Overall, these findings demonstrated that modified mRNA could be used to produce high levels of proteins of interest while avoiding strong inflammatory responses. The use of pseudouridine and ARCA considerably improves the translation of modRNA (chemically modified mRNA), decreases its immunogenicity in vitro, and boosts the yield per reaction. It appears to be the best option for dealing with clinical problems (Hadas et al. 2019; Janowski and Andrzejewska 2022).

## Innovations in mRNA delivery for effective therapeutics

After creating a useable vaccine as a medical agent, mRNA molecules must reach specific target cells and produce enough proteins of interest to have therapeutic effects. The mRNA is quite fragile. The rapid fragmentation of mRNA by environmental and internal enzymes makes lab work challenging and mRNA transfer to cells intimidating. Additionally, because mRNA strands are long and negatively charged, they cannot easily pass through the cell’s protective lipid membranes. It is possible to complete this task in a variety of ways. Tiny balls of fat called lipid nanoparticles, or LNPs in particular, have been extensively studied and successfully used in the clinic to transport small compounds, siRNA medicines, and mRNA (Akinc et al. 2019; Baden and Hana 2021; Anderson et al. 2020; Polack et al. 2020). Particularly, the mRNA-1273 (Moderna) (Baden and Hana 2021; Anderson et al. 2020) and BNT162b (BioNTech) (Polack et al. 2020) coronavirus disease 2019 (COVID-19) vaccines use lipid nanoparticles to deliver antigen mRNA. The neutral phospholipid, cholesterol, polyethylene-glycol (PEG)-lipid, and an ionizable cationic lipid are the four primary components of the LNPs in mRNA COVID-19 vaccines. In order to interact with the anionic mRNA during particle formation and to assist membrane fusion during internalization, the latter contains positively charged, ionizable amine groups (at low pH) (Evers et al. 2018; Reichmuth et al. 2016). The mRNA is packaged in thousands of these four components, which also protect it from harmful enzymes and transport it into cells where it is released and used to generate proteins. Although the idea seems obvious, perfecting it was far from easy.

## Discussion

The discovery and understanding of nucleic acids, particularly mRNA, have had a profound impact on human history and have revolutionized the field of medicine. The exploration of mRNA began in the mid-twentieth century and has since progressed rapidly, leading to significant breakthroughs and advancements in various areas, including vaccine development.

The COVID-19 pandemic served as a catalyst for the widespread recognition and utilization of mRNA technology in vaccine development. Teams from Moderna, BioNTech, and CureVac harnessed the potential of mRNA to encode viral antigens and trigger an immune response. This approach represented a departure from traditional vaccine development methods and offered several advantages, such as rapid manufacturing, adaptability to new virus strains, and excellent safety profiles.

The success of mRNA-based COVID-19 vaccines, such as those developed by Pfizer-BioNTech and Moderna, can be attributed to the visionary work of scientists and researchers in the field. Individuals like Katalin Karikó, Drew Weissman, Edward Darzynkiewicz, Robert Rhodes, Ugur Sahin, and Ozlem Tureci have made significant contributions to mRNA research, paving the way for the development of effective vaccines and therapies.

The engineering of cap analogs and nucleotide modifications has greatly enhanced the translation efficacy and stability of mRNA, further propelling its potential as a powerful tool in medicine (Jurga and Barciszewski 2022). The collaboration between researchers, such as Robert Rhodes and Edward Darzynkiewicz, in developing cap modifications and stabilizing mRNA molecules has played a crucial role in unlocking the translational efficiency and immunogenicity of mRNA.

The story of mRNA is one of remarkable progress, fueled by a series of breakthroughs and collaborations. The success of mRNA vaccines against COVID-19 has not only saved countless lives but has also opened up new possibilities for the future of medicine. The speed and effectiveness with which mRNA-based vaccines were developed and deployed during the pandemic highlight the tremendous potential of this technology.

Looking ahead, ongoing research in mRNA technology holds immense promise for developing vaccines against other infectious diseases and for treating various genetic and rare disorders. The lessons learned from the COVID-19 pandemic and the pioneering work of scientists like Ugur Sahin and Ozlem Tureci have paved the way for the development of new vaccines and therapies that can target a wide range of diseases and medical conditions.

In conclusion, the discovery and exploration of mRNA have revolutionized the field of medicine, and its potential is only beginning to be realized. The remarkable achievements made in mRNA research, particularly in the context of COVID-19 vaccine development, highlight the power of collaboration, visionary thinking, and scientific innovation. The future of medicine looks brighter than ever as we continue to unlock the full potential of this incredible molecule.

## Future of mRNA vaccines

The future of mRNA vaccines is poised for remarkable progress, with ongoing research and clinical trials unveiling a landscape of possibilities for combating infectious diseases. The current landscape of mRNA vaccines is dynamic and rapidly evolving. In a comprehensive global evaluation, approximately 90 lead developers of mRNA vaccines have been identified. These pioneering developers collectively have an impressive total of 137 mRNA vaccine candidates actively in the pipeline (Kumar et al. 2022). This robust portfolio of mRNA vaccine candidates is distributed across various stages of development, highlighting the versatility and potential of this groundbreaking technology. Notably, a substantial 76% of these candidates are in the preclinical or exploratory phase, representing the innovative spirit and continuous exploration of novel vaccine targets and strategies.

At the forefront of this exciting frontier, multiple mRNA vaccines are advancing steadily, some even reaching the coveted phase 3 trials. One particularly noteworthy advancement is the mRNA cytomegalovirus (CMV) vaccine, which has gracefully entered its phase 3 stage after showcasing promising results in phase 2. Notably, in participants who were previously seronegative for CMV, the vaccine demonstrated a substantial increase in neutralizing antibody titers against epithelial cell infection (Moderna Announces Clinical Progress From Its Industry-Leading mRNA Vaccine Franchise and Continues Investments to Accelerate 2021). This encouraging data hints at the vaccine’s potential to tackle CMV, a virus that can have significant health implications, particularly in vulnerable populations.

In the realm of influenza, Moderna’s mRNA-based influenza vaccine has commenced phase 3 trials after a reassuring phase 2 journey (Moderna Announces Positive Interim Phase 1 Data for mRNA Flu Vaccine and Provides Program Update, n.d.). The vaccine’s capacity to elevate geometric mean titers (GMTs) against H1N1 and H3N2 strains has generated optimism among researchers and healthcare professionals. Equally promising, no significant safety concerns emerged in the preliminary analysis up to day 29, bolstering confidence in its safety profile.

Moreover, the latest insights from the phase 3 mRNA respiratory syncytial virus (RSV) vaccine trial are equally compelling, showcasing an impressive vaccine efficacy of 83.7% against RSV. This remarkable result is a testament to the potential of mRNA vaccines to tackle formidable respiratory infections effectively. With plans in place for regulatory approval, this mRNA RSV vaccine could soon join the arsenal of tools in our fight against respiratory diseases.

Promisingly, research continues to thrive at earlier stages of development, with some vaccines in phase 1 and 2 trials showing immense potential. The mRNA-4157/V940 vaccine, a collaborative effort between Moderna and Merck, has revealed a groundbreaking 44% reduction in the risk of melanoma recurrence or death in a phase 2 trial. This milestone underscores the versatility of mRNA vaccine technology in addressing complex health challenges beyond infectious diseases (Moderna and Merck Announce mRNA-4157/V940, an Investigational Personalized mRNA Cancer Vaccine, in Combination With KEYTRUDA(R) (Pembrolizumab), Met Primary Efficacy Endpoint in Phase 2b KEYNOTE-942 Trial, n.d.).

Preclinical animal studies have also illuminated the extraordinary capabilities of mRNA vaccines. For instance, the mRNA Epstein-Barr virus (EBV) vaccine elicited antibody levels surpassing those observed in naturally infected human sera (Rozman et al. 2022) These compelling findings ignite hope for innovative strategies to prevent and manage EBV-related conditions.

The remarkable developments in mRNA vaccine research have transcended boundaries, instigating interest in applying this technology to combat a spectrum of infectious diseases. One such endeavor is the ongoing preclinical research funded by the National Institutes of Health (Hayashi et al. 2022), exploring the potential of mRNA vaccines in addressing the global challenge of malaria.

In summation, the future of mRNA vaccines is not only bright but also transformative. As research continues to unfold and as more vaccines progress through clinical trials, we stand on the cusp of a new era in vaccine development and disease prevention, driven by the potential of mRNA technology to redefine our approach to public health challenges.
